# Virus attacks fish by muscling its way into cells

**DOI:** 10.7554/eLife.107508

**Published:** 2025-06-02

**Authors:** Ping-Ping Liu, Zhe Wei, Xian-Wei Wang

**Affiliations:** 1 School of Life Sciences, State Key Laboratory of Microbial Technology, Shandong University Qingdao China; 2 Laboratory for Marine Biology and Biotechnology, Qingdao Marine Science and Technology Center Qingdao China

**Keywords:** myosin light chain 3, nervous necrosis virus, receptor, macropinocytosis, Viruses

## Abstract

Nervous necrosis virus typically enters host cells via endocytosis, but it can also enter via a process called macropinocytosis.

**Related research article** Yao L, Zhang W, Yang X, Yi M, Jia K. 2024. Myosin light chain 3 serves as a receptor for nervous necrosis virus entry into host cells via the macropinocytosis pathway. *eLife* **13**:RP104772. doi: 10.7554/eLife.104772.

Viruses are tiny invaders that need to get inside host cells to survive and reproduce. But entering a cell isn't as simple as knocking on the door – viruses have to trick specific proteins on the cell surface in order to gain entry. Figuring out which proteins are used by viruses to enter cells could, therefore, help researchers to develop new ways to block viruses and prevent the spread of the diseases they cause.

Nervous necrosis virus (NNV) is a small RNA virus that primarily infects marine fish, causing a deadly disease called viral nervous necrosis. This disease can wipe out entire fish populations in aquaculture farms, causing huge financial losses (Costa and Thompson, 2016). NNV relies on its capsid protein to bind to receptors on the surface of host cells, thereby facilitating cellular invasion (Munday et al., 2002).

It is well known that NNV can enter cells through a process called clathrin-mediated endocytosis, which involves the capsid protein – the protein shell that encloses the genetic material of the virus – binding to various receptors (Chang and Chi, 2015; Zhang et al., 2020). However, viruses are masters of deception, and NNV can also enter cells via a process called macropinocytosis (Liu et al., 2005), although the identities of the receptors involved in this process have been a mystery.

Now, in eLife, Kuntong Jia, Meisheng Yi and colleagues at Sun Yat-sen University – including Lan Yao and Wanwan Zhang as joint first authors – report that a protein called MmMYL3 serves as a cell surface receptor that NNV can exploit to enter host cells via macropinocytosis (Yao et al., 2024). The virus binds to MmMYL3 – which is best known for its role in muscle contraction – to activate a signaling pathway called the IGF1R-Rac1/Cdc42 pathway (Figure 1).

**Figure 1.**
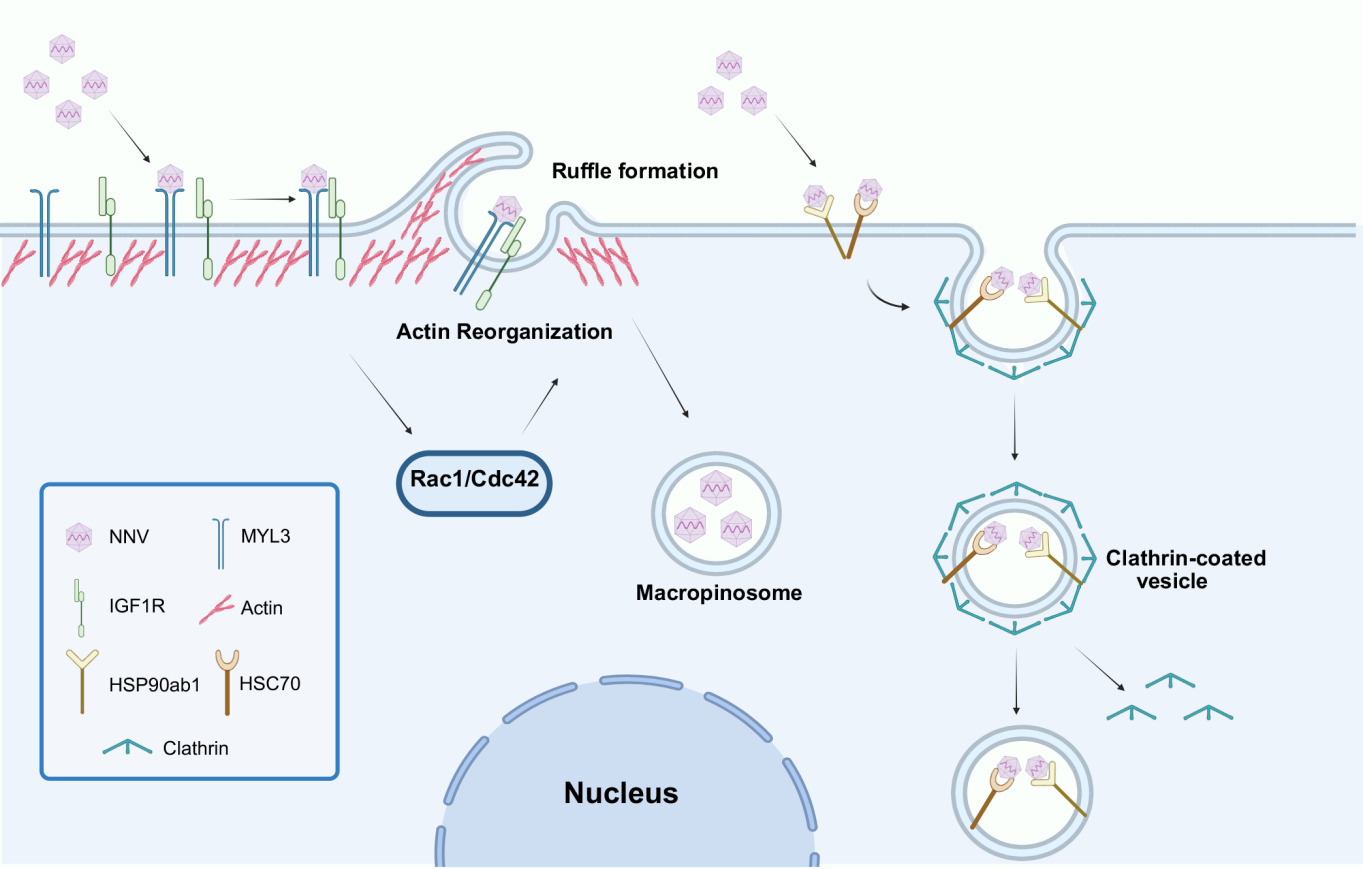
Nervous necrosis virus (NNV) can enter host cells via two distinct pathways. It is well established that NNV can enter host cells by binding to various receptors (such as the heat shock proteins HSP90ab1 and HSC70), which leads to the formation of clathrin-coated vesicles inside the cell (right). Now Yao et al. report that NNV can also enter host cells by binding to a protein called MmMYL3 (short for myosin light chain 3 of marine medaka; left). This interaction activates a receptor called IGF1R and two small GTPase proteins (Rac1 and Cdc42), resulting in a reorganization of the actin cytoskeleton and the formation of ruffles in the cell membrane (top). These ruffles become vesicular structures inside the cell called macropinosomes that contain the virus, along with extracellular fluid and particles. IGF1R: insulin-like growth factor receptor 1.

This figure was created using BioRender.com.

Yao et al. started by using various lab techniques – including co-immunoprecipitation, pull-down assays, and surface plasmon resonance – to confirm that MmMYL3 binds specifically to the ARM and S domains of the capsid protein. They also showed that MmMYL3 sits on the cell surface, making it an easy target for the virus. Receptor blocking experiments, including antibody blocking and protein competition assays, demonstrated that treating cells with MmMYL3 antibodies, or with peptides that resembled the ARM domain, effectively suppressed NNV invasion.

Pre-incubating NNV with recombinant MmMYL3 protein also inhibited viral attachment to host cells. Moreover, receptor reconstruction experiments conducted in cells that were insensitive to NNV confirmed that MmMYL3 is indeed an essential receptor for the virus. In addition, in vivo experiments performed using the species marine medaka showed that pre-treating NNV with recombinant MmMYL3 protein significantly improved the survival rate of infected fish, thus validating the functional importance of MmMYL3 in NNV infection at the organismal level. These findings all suggest that this protein can serve as a new receptor for NNV.

Yao et al. then dug deeper into the signaling pathway that is activated by the virus binding to MmMYL3 (Figure 1). The next step involves the MmMYL3 protein binding to a different receptor called IGF1R. This sets off a chain reaction involving two small signaling proteins, Rac1 and Cdc42, which reorganize the cytoskeleton of the cell, and allow the cell membrane to form large vesicles that engulf the virus. Such vesicles are a hallmark of macropinocytosis (Mercer and Helenius, 2009).

Interestingly, MmMYL3 is not the first myosin protein to be identified as a viral receptor. Previous studies have shown that non-muscle myosin IIA acts as a receptor for herpes simplex virus (Arii et al., 2010), while myosin heavy chain 9 facilitates the entry of porcine reproductive and respiratory syndrome virus (Li et al., 2018). This suggests that myosin proteins may serve as a broader family of viral entry receptors than previously thought.

What makes MmMYL3 particularly intriguing is its dual role – a protein traditionally associated with muscle function is now implicated in viral entry, illustrating how viruses can repurpose host proteins to suit their needs. This discovery opens up new possibilities for antiviral strategies. By targeting MmMYL3 or the associated signaling pathways, it might be possible to develop therapies that block viral entry without affecting normal cellular functions.
